# Selective transmission of some HIV-1 subtype C variants might depend on Envelope stimulating dendritic cells to secrete IL-10

**DOI:** 10.1371/journal.pone.0227533

**Published:** 2020-01-24

**Authors:** Evelyn Ngwa Lumngwena, Bianca Abrahams, Liliwe Shuping, Claudia Cicala, James Arthos, Zenda Woodman

**Affiliations:** 1 Department of Medicine, Faculty of Health Sciences, University of Cape Town, Cape Town, South Africa; 2 Institute of Infectious Disease and Molecular Medicine, Faculty of Health Sciences, University of Cape Town, Cape Town, South Africa; 3 Institute for Medical Research and Medicinal Plants studies (IMPM), Ministry of Scientific Research and Innovation (MINRESI), Yaounde, Cameroon; 4 Department of Integrative Biomedical Sciences, Faculty of Health Sciences, University of Cape Town, Cape Town, South Africa; 5 National Institute for Communicable Diseases, National Health Laboratory Services, Johannesburg, South Africa; 6 National Institute of Allergy and Infectious Diseases (NIAID), NIH, Bethesda, Maryland, United States of America; Instituut voor Tropische Geneeskunde, BELGIUM

## Abstract

Envelope (Env) phenotype(s) that provide transmitted founders (TF) with a selective advantage during HIV-1 transmission would be the ideal target for preventative therapy. We generated Env clones from four individuals infected with a single virus and one participant infected with multiple variants at transmission and compared phenotype with matched Envs from chronic infection (CI). When we determined whether pseudovirus (PSV) of the five TF and thirteen matched CI Env clones differed in their ability to 1) enter TZM-bl cells, 2) bind DC-SIGN, and 3) *trans*-infect CD4+ cells there was no association between time post-infection and variation in Env phenotype. However, when we compared the ability of PSV to induce monocyte-derived dendritic cells (MDDCs) to secrete Interleukin-10 (IL-10), we found that only TF Envs from single variant transmission cases induced MDDCs to secrete either higher or similar levels of IL-10 as the CI clones. Furthermore, interaction between MDDC DC-SIGN and Env was required for secretion of IL-10. When variants were grouped according to time post-infection, TF PSV induced the release of higher levels of IL-10 than their CI counterparts although this relationship varied across MDDC donors. The selection of variants during transmission is therefore likely a complex event dependent on both virus and host genetics. Our findings suggest that, potentially due to overall variation in N-glycosylation across variants, nuanced differences in binding of TF Env to DC-SIGN might trigger alternative DC immune responses (IRs) in the female genital tract (FGT) that favour HIV-1 survival and facilitate transmission.

## Introduction

The results of the Thai RV144 and the Merck STEP vaccine trials highlight the need for a thorough understanding of the dynamic inter-relationship between host immune responses (IRs) and HIV-1 immune escape and pathogenesis [1,2]. Dendritic cells (DCs) are central to the development of IRs by interacting with the innate immune system and triggering adaptive responses [3]. It has been suggested that DCs directly facilitate HIV-1 transmission by either becoming infected, internalising virus via Dendritic Cell-Specific Intercellular adhesion molecule-3-Grabbing Non-integrin (DC-SIGN) for *trans*-infection of CD4 T cells [4,5] and/or modulating IR to HIV-1 [6]. Therefore, DCs may be instrumental in establishing productive HIV-1 clinical infection especially as they make first contact with incoming virus in the female genital tract (FGT).

DCs have several regulatory functions, each mediated by alternative signals. They interact with and guide the responses of the innate immune system, but also initiate adaptive and tolerogenic responses [3]. Regulatory DCs (DCregs), which are immunosuppressive, promote tolerance, T cell anergy and potentially, facilitate viral infection [7]. It is thus not surprising that Herpes, Papilloma and Foot-and-mouth disease viruses are known to manipulate DC function either via direct infection or by immune regulation to promote their own survival [8]. HIV might also have evolved to regulate DC-mediated responses as infection is associated with reduced expression of DC co-stimulatory molecules CD80 and CD86 and impaired DC maturation [5,9]. Furthermore, human monocytes expressed high levels of interleukins (IL) -1, -6, -10,-18, -23, -27, CCL2, 4, 20, CXCL2, 13, TSLP and TNFα in response to gp120 stimulation and the authors suggested that Env could induce a “cytokine burst” [10]. Another, earlier study showed that HIV-1 Envelope (Env) binding to DC-SIGN via its high mannose (HM) N-glycans also induced monocyte-derived DCs (MDDCs) to secrete IL-10 [9]. IL-10, an anti-inflammatory cytokine, limits Th1 expansion by inhibiting DC IL-12 production and regulating chemokine expression [11,12]. It also promotes the emergence of DCregs, inhibits T cell function [7,9] and induces the emergence of regulatory T cells (Treg), Tr1 cells, and tolerogenic DCs [13]. Therefore, the presence of IL-10 soon after transmission may trigger DC-mediated IRs that help HIV survival and infection. However, only specific Env clones induced MDDCs to secrete IL-10 *in vitro* [9], suggesting that only some variants might be able to deregulate DC IRs.

As DC-SIGN binds to Env N-glycans, and differences in Env N-glycosylation have been linked to HIV-1 transmission, we hypothesised that variation in Env HM N-glycans could be a determining factor in the ability of virus to induce MDDCs to release IL-10. For example, transmitted founders (TFs) tend to carry fewer potential N-glycan sites (PNGs) [14,15] that are enriched with HM N-glycans when compared to variants from chronic infection [16]. The loss and gain of N-glycans at specific Env positions alter binding to CD4, CCR5, α_4_β_7_ and DC-SIGN [17–19] and Nabatov *et al*. (2008) reported that variation in Env N-glycans caused alterations in interactions with DC-SIGN [20]. DC-SIGN was shown to bind to PNGs at positions N276, N295, N356, N386 and N392 of Env [21,22] and it was also reported that DC-SIGN interacted with HM N-glycans with different affinity due to the number of mannose residues [23]. Therefore, the presence or absence of specific PNGs known to carry HM could result in nuanced changes in the binding of TF Env to DC-SIGN, triggering IRs that favour HIV-1 transmission. In this report we find that even though TF Envs are not enriched with N276, N295, N356, N386 and N392, they induced MDDCs to secrete high levels of IL-10 via interaction with DC-SIGN. This has important implications for HIV prevention strategies as previous studies had found that blocking IL-10 improved the efficacy of a DNA vaccine in other viral infections in other models [24].

## Methods

### Ethics statement

Buffy coats were obtained from the Western Province blood bank and as all donors were anonymous, there was no need to obtain informed consent. The protocol was approved by the University of Cape Town Ethics in Research Committee of the Faculty of Science, SFREC 003_2012.

### Samples

HIV-negative women were tracked longitudinally and tested for HIV infection using enzyme immunoassay assay, p24 antigen test, rapid HIV tests and/or RNA PCR. The time relative to infection was confirmed by Fiebig staging and infection with either single or multiple subtype C variants was determined by single-genome-amplification (SGA) [25].

Full-length *envelope* (*env*) from five HIV positive women sampled during acute infection (AI) 2–5 weeks post-infection (wpi) with follow up longitudinal samples collected 2–3 years post-infection (ypi) were used for this study (Accession numbers: JX976722.1, KC863497.1, FJ443390.1, 47:KC833437.1, KJ700457.1, DQ435683.1, KC863280.1, KC863276.1, FJ443142.1, HQ625601, HQ625600.1, FJ443357.1, HQ625599.1, KC863388.1, HQ625599.1 (www.ncbi.nlm.nih.gov). Eighteen functional Env clones (numbered C1 –C18) were generated from SGA-derived PCR products, cloned into pcDNA/his-topo (Invitrogen) or pTarget mammalian expression vectors (Promega). All functional clones from the five participants at acute infection were identical to the consensus of 16–23 SGA-derived sequences at the earliest time-point. Due to high diversity at 2–3 ypi (range = 1.1–3.18%) Envs were cloned from 10–23 SGA chronic infection sequences that were most closely related to consensus and represented the conserved N-glycan profile of the dominant viruses at that time point. PNG frequency analysis was carried out on 36 matched Env consensus sequences from acute and chronic infection (accession numbers: (JX976651.1, FJ443216.1,FJ443182.1, FJ443984.1, KC894137.1, KC863412.1, JN681229.1, KC863558.1, KC863493.1, KC863519.1, KC863542.1, KC833437.1, HQ625599.1, KC863410.1, JX976681.1, HQ625602.1, KC863457.1, HQ625605.1, FJ443279.1, FJ443963.1, KF996701.1) and random subtype C sequences (www.ncbi.nlm.nih.gov).

### Cell culture

Human embryonic kidney (HEK) 293T [Kind gift from Carolyn Williamson, Institute of Infectious Diseases and Molecular Medicine, University of Cape Town and National Health Laboratory Services (NHLS)] and TZM-bl cells [NIH AIDS Reagent Program (ARP), Division of AIDS, NIAID from Dr. John C. Kappes, Dr. Xiaoyun Wu and Tranzyme Inc] were maintained in Dulbecco modified Eagle high glucose medium (DMEM) (Lonza, Whitehead Scientific) supplemented with 10% fetal bovine serum (FBS) (PAA, Biocom Biotech), 1 U ml^−1^ penicillin and 1 μg ml^−1^ streptomycin (Lonza, Whitehead Scientific). Raji cells (NIH-ARP from Alexandre Kabamba at the National Institute of Communicable disease (NICD), South Africa) and Raji DC-SIGN cells (NIH-ARP, from Drs. Li Wu and Vineet N. KewalRamani) [26] were maintained in RPMI supplemented with 10% FBS. All cells were grown in a humidified incubator at 37 ^o^C with 5% CO_2_

### Pseudovirus production

To produce pseudovirus (PSV), HEK 293T cells were transfected with 2.5 μg of gp160 and 5 μg of the viral backbone (pSG3ΔEnv) mixed in a 1:3 ratio with PEI transfection reagent (Sigma-Aldrich, USA). Culture medium was collected 48 hr following transfection, clarified using a 0.22 μm pore size filter, FBS was adjusted to 20% and PSV stored in single-use aliquots at -70°C.

### p24 ELISA

To determine the concentration of PSV, a chemiluminescent p24 ELISA (Aalto Bio-reagents) and the TROPIX® detection system (CDP-Star®, Applied Biosystems) was used. Sheep anti-HIV-1 p24 gag antibody (Aalto Bio-reagents) diluted to 3 μg ml^−1^ in coating buffer (0.1 M NaHCO_3_; pH 8.5) was used to capture PSV inactivated for 1 hr with 1% Empigen-TBS, pH 7.4. A mouse anti-HIV-1 p24 antibody (Aalto Bio-reagents) conjugated to alkaline phosphatase and TROPIX® chemiluminescent substrate for alkaline phosphatase with enhancer (CSPD) detection reagent (Life technologies) was used to detect luminescence using a plate reader (Turner Biosystems, ANATECH Instruments). Relative light units (RLU) were converted to p24 concentration (ng ml^−1^) using a standard curve and non-linear regression analysis.

### Pseudovirus entry assay

To determine pseudovirion entry efficiency, TZM-bl cells [27,28] were seeded at 10^4^ cells per well before infection with 5-fold serial dilutions of PSV for 48 hr at 37°C. All PSV were normalised to 100 ng ml^-1^ p24 prior to serial dilution and infectivity was measured by luminescence using BriteGlo® (Promega) and luminometer (Turner Biosystems®). Negative controls included background luminescence of wells carrying cells only or cells transfected with pSG3ΔEnv only.

### DC-SIGN binding assay

A DC-SIGN binding assay was adapted from Alexandre *et al*. (2012) [29]. Briefly, 10 ng p24 pseudovirus was added to Raji and Raji DC-SIGN cells at a density of 10^5^ cells/well in a total volume of 200 μl. After binding for 2.5 hr at 37°C, cells were washed four times by centrifugation (2500 rpm) with RPMI to remove all unbound virus, then lysed in 1% (v/v) Empigen-TBS before determining cell-associated p24 (Aalto Biosystems).

### *Trans*-infection

Pseudovirus was bound to 10^5^ Raji DC-SIGN cells or 5 X10^3^ MDDCs as outlined above before adding to TZM-bl cells seeded at a density of 10^4^ cells/well. Infectivity was allowed in a 37 ^o^C incubator with 5% CO_2_ for 48 hr and PSV entry was measured using BriteGlo® (Promega) and luminometer (Turner Biosystems® Modulus Microplate).

### Recombinant gp140 preparation

C12, C13 and C14 *envs* were commercially synthesized from the C-terminal end of the signal peptide to the transmembrane domain (residues 31 to 680 HXB2 numbering, (Mutagenex, Piscataway, NJ), codon-optimized (DNA2.0, Menlo Park, CA) and the endogenous Env signal peptide was replaced with that of the tissue plasminogen activating protein (TPA). Non-adherent Chinese hamster ovary (CHO-S) cells (Invitrogen, Grand Island, NY) were transfected using the FreeStyleTM MAX expression medium (Gibco, 12651–014, USA) according to the manufacturer’s instructions in a 37 ^o^C incubator on a rotator. Cell culture medium was harvested 72 hr post transfection by centrifugation at 2000 rpm for 10 min, filtered through a 0.22 μm filter (Millipore, USA), passed over a column of *Galanthus nivalis* lectin sepharose (Vector Labs, Burlingame, CA) and gp140 was eluted with 20 mM Glycine-HCl, pH 2.0, 150 mM NaCl, 500 mM α-methyl-mannopyranoside (Sigma, St. Louis MO). SDS PAGE and Western blotting was used to identify peak fractions which were pooled and then concentrated with a stir cell concentrator (Millipore, Billerica, MA). The protein was then dialysed against HEPES, pH 7.4, 150 mM NaCl and quantitated by absorbance at O.D. λ280 (extinction coefficient 1.1) and bicinchoninic acid protein assay (Thermo Scientific, Rockford, IL).

### Expression and incorporation of Envelope

After HEK 293T cells were transfected with C12, C13 and C14 gp160 in the presence and absence of pSG3Δenv, the culture medium was harvested to isolate PSV and the cells lysed with RIPA buffer [10 mM Tris, pH 7.2, 2 mM EDTA, pH 8.0, 150 mM NaCl_2_, 1% Triton X-100 (Sigma-Aldrich) and 0.1% phenylmethylsulfonyl fluoride (PMSF)] to determine cell-associated Env. The culture medium was layered onto a 20% glycerol cushion and ultracentrifuged at 26000 rpm for 2 hr at 4°C. The supernatant was removed and the PSV resuspended in the remaining supernatant. Cell lysates and PSV were separated by SDS PAGE before Western Blotting with sheep anti-gp120 and antibodies against actin and p24, respectively.

### Generation of monocyte derived dendritic cells (MDDCs)

PBMCs were obtained from buffy coats by density gradient centrifugation using Ficoll-Hypaque (Sigma-Aldrich). Monocytes were isolated from PBMCs by positive selection using CD14+ coated beads (130-050-201 Miltenyi, USA or Biochom Biotech, S.A) according to manufacturer’s instructions or by adherence. Monocytes were adhered in serum-free medium for 2 hr at 37 ^o^C with 5% CO_2_ before non-adherent cells were removed by washing. Isolated monocytes were differentiated in medium with 1000 U ml^-1^ GM-CSF (PHC2013, BioSource) and 500 U ml^-1^ of recombinant human IL-4 (PHC0045, BioSource) for six days with recombinant cytokines supplemented every other day.

### MDDC stimulation

PSV titre was determined by infecting TZM-bl cells for 48 hr before measuring luminescence. PSV, diluted to a concentration that increased luminescence to 50 X background was used in all stimulation experiments. Day six MDDCs were washed with PBS to remove all differentiation medium and either titred PSV (50 X background) or purified gp140 at a concentration of 2 μg ml^-1^ were added to 2 x 10^6^ cells ml^-1^. Stimulation was done in culture medium containing 2% (v/v) AB human serum (H1513, Sigma), 1% P/S (v/v) (Sigma Aldrich), 1% non-essential amino acids (Gibco-Life technologies, USA), 1% (v/v) sodium pyruvate (Gibco, USA) and 1% (v/v) L-glutamine (Sigma Aldrich) in RPMI for 24 hr. Following stimulation, the plates were centrifuged at 2000 rpm for 10 min to pellet any cells in suspension and the culture supernatants harvested and stored in single-use aliquots at -80 ^o^C.

### Luminex multiplex assay for cytokine quantification

The level of cytokines released by MDDCs into the culture medium was quantified using Luminex suspension array technology with cytokine-specific antibody-immobilized magnetic beads (Millipore, USA). Biotinylated secondary antibodies and streptavidin-phycoerythrin conjugates were used to detect bound cytokines using a Luminex platform (Bio-rad, USA) to determine the Mean Fluorescence Intensity (MFI). In order to quantify the level of cytokines, a standard curve was used to determine protein concentration relative to MFI. Linear regression was used to determine the concentration of cytokines in each experimental sample.

### IL-10 ELISA

The levels of IL-10 were also measured by ELISA (Quantikine, R & D Systems) based on a quantitative enzyme immune-assay. In this assay, a monoclonal anti-IL-10 specific antibody was used to pre-coat the ELISA plate, followed by addition of samples and the amount of IL-10 was measured by addition of enzyme-linked polyclonal anti-IL-10 antibodies. Standards and colorimetry was used to determine quantitative differences between samples.

### Statistical analysis

Mann-Whitney two-tailed comparison was used to compare non-paired events and Wilcoxon signed rank test was used for pairwise comparison of paired events (GraphPad Prism 5.0). Mean or median values obtained from triplicates of 2–4 biological repeats, where indicated, were compared and a *P*-value < 0.05 was considered as statistically significant. Correlation analysis was done using two-tailed Spearman test. The step-down false discovery rate to take into account Type I errors was used to adjust for multiple comparisons.

## Results

### Env entry efficiency, DC-SIGN binding and CD4+ cell *trans*-infection

**Fig 1:**
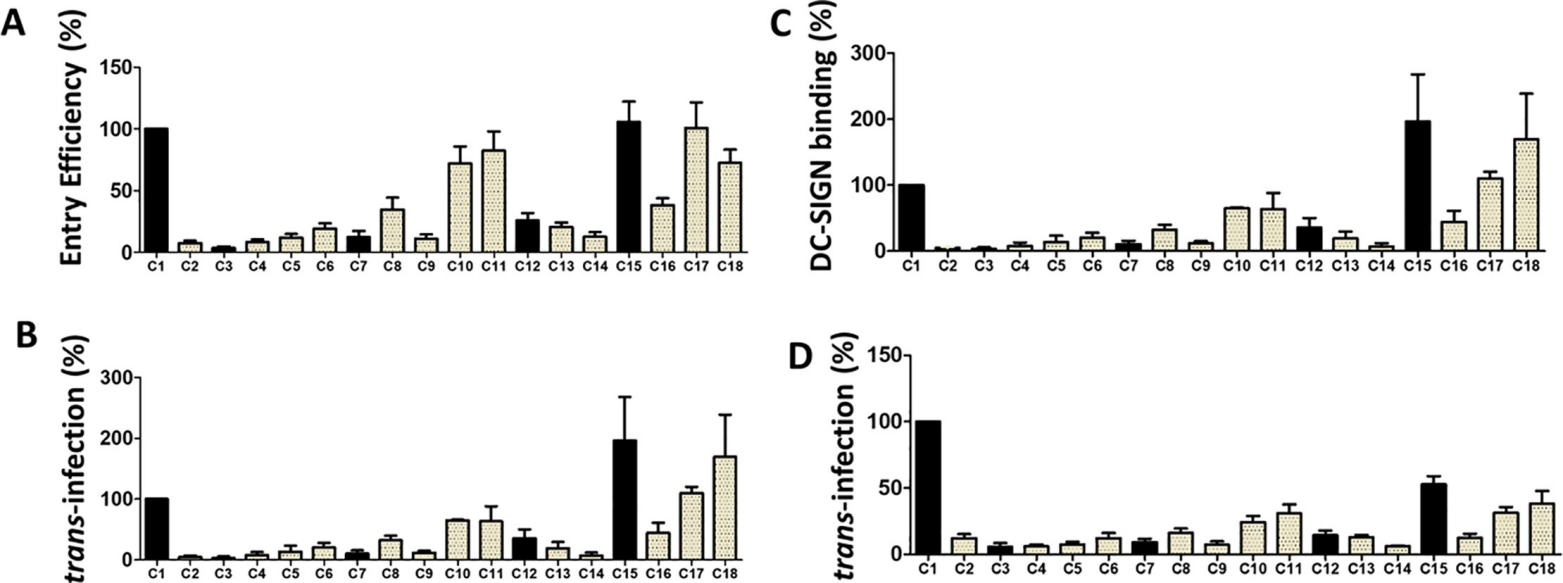
Characterisation of Envelope phenotype. A) Pseudovirus (PSV) equivalent to 100 ng ml^−1^ p24 were compared for their ability to enter TZM-bl cells. Entry was monitored through the activation of a luciferase reporter gene under the control of the LTR. The bar graphs are means of four biological repeats with error bars showing standard error of the mean (SEM). Statistical analysis of the medians was done using Mann Whitney two tailed comparison. B) PSV equivalent to 10 ng p24 were bound to Raji-DC-SIGN cells and after washing the cells, bound PSV was detected by p24 ELISA and normalized to input PSV. Mean and SEM of two biological repeats are indicated. C) Raji-DC-SIGN cells or D) monocyte derived dendritic cells (MDDCs) were used to *trans*-infect TZM-bl cells. The averages of two C) and four D) independent repeats with SEM are indicated. To compare data across biological repeats, all raw values are indicated as a percentage of C1.

Eighteen matched subtype C Envs were selected as they represented variants infecting five participants at 2–5 wpi and 2–3 ypi of a high risk cohort in South Africa [30]. To determine whether TF variants preferentially carried Env with enhanced function we determined whether Envs varied in TZM-bl entry efficiency, binding to DC-SIGN and *trans*-infection of CD4+ cells. As expected, PSV entry efficiency varied 28 –fold, confirming the high variation in Env function across variants (Fig 1A). PSV DC-SIGN binding varied 5-fold possibly because the PNGs suggested to comprise the DC-SIGN binding site [21,22] were not conserved across the clones (Table 1). However, the frequency of N276, N295, N356, N386 and N392 and combinations thereof were not linked to differences in DC-SIGN binding, suggesting that these PNGs were not required for optimal binding of the Envs in this study (Fig 1B).

**Table 1:** Characterisation of Envelope clones and conservation of potential N-glycan sites (PNGs).

Participant ID	ID	Time post infection (wks)	No. of PNGs	Position of N-Glycan sites							
				241	262	N276	N295	N356	N386	N392	448
CAP45	C1	2	29	+	+	+	-	+	+	-	-
	C2	108	28	+	+	-	-	+	+	-	-
CAP177	C3	2	24	+	+	+	-	-	+	-	+
	C4	173	33	+	+	+	-	-	+	-	+
	C5	173	31	+	+	+	-	-	+	-	+
	C6	173	35	+	+	+	-	-	+	-	+
CAP206	C7	4	25	+	+	+	-	-	+	-	-
	C8	173	28	+	+	+	-	-	-	+	-
	C9	173	27	+	+	+	-	-	-	-	-
	C10	173	27	+	+	+	-	-	+	+	-
	C11	173	26	+	+	+	-	-	+	-	-
CAP210	C12	2	33	+	+	+	-	+	+	+	+
	C13	80	32	+	+	+	-	+	+	+	-
	C14	80	33	+	+	+	-	+	+	+	-
CAP 239	C15	5	29	+	+	-	+	+	+	+	+
	C16	173	31	+	+	+	-	+	+	+	+
	C17	173	32	+	+	+	-	+	+	+	+
	C18	173	30	+	+	+	-	+-	+	+	+
Clonal frequency^a^ (%)				100	100	89	6	50	89	50	50

Presence and absence of PNGs analysed in this study are indicated with plus and minus signs, respectively.

^a^Frequency of PNGs in the 18 functional clonal sequences used in this study

When Raji-DC-SIGN cells and MDDCs were used to bind PSVs, *trans-*infection of TZM-bl cells varied 48—and 16—fold, respectively (Fig 1C and 1D) and there was a strong correlation between the two methods (*P* = .0001, r = .8762) (S1D Fig). Whereas PSV EE was significantly associated with *trans*-infection (P = .0001, r = .9525), irrespective of whether pseudovirus was first bound to Raji cells or MDDCs (S1A and S1B Fig), a correlation was only found between DC-SIGN binding and *trans*-infection when Raji-DC-SIGN cells were used as the vehicle (S1C Fig). This is likely due to MDDC receptors other than DC-SIGN interacting with Env [20]. Overall, correlation analysis indicated that PSV *trans*-infection is dependent on DC-SIGN binding and Env entry efficiency, confirming previous studies (S1A Fig) [17,31–33].

Phenotypic comparison between TF and matched CI clones showed that only C1 and C12 had consistently higher entry efficiency, DC-SIGN binding and *trans*-infection than matched CI PSV (Fig 1A–1D). Given the paucity of CD4+ T cells in the FGT, it has been suggested that those Envs best able to interact with CD4, CCR5 and DC-SIGN are selectively transmitted. However, our findings suggest that successful HIV-1 transmission might not depend on the magnitude with which TF Envs mediate host cell entry of CD4+ cells, DC-SIGN binding and *trans*-infection but rely on another, as yet undefined, mechanism.

### Env stimulation of MDDC Interleukin-10 secretion

It was previously shown that binding of highly mannosylated gp120 to a MDDC mannose C type lectin receptor stimulated the release of IL-10 [9]. Go *et al*. (2011) showed that TF Envs were enriched with HM N-glycans at N241, N262, N386, N392 and N448 [16]. As we have previously shown that these sites are important for C15 and C16 Env function [34], we selected nine PSV clones because they carried these PNGs at different frequencies to determine whether the presence or absence of the N-glycan sites could explain differences in IL-10 secretion (Table 1). An important approach of this study was to compare TF from single variant transmission cases to matched CI clones as we hypothesized that transmission of multiple variants were less likely to have undergone selection. Therefore, when CAP177 was shown to be infected by two distinct variants [35], the participant was included in the study to determine whether C3 and C4 also differed in their ability to stimulate IL-10 release.

**Fig 2:**
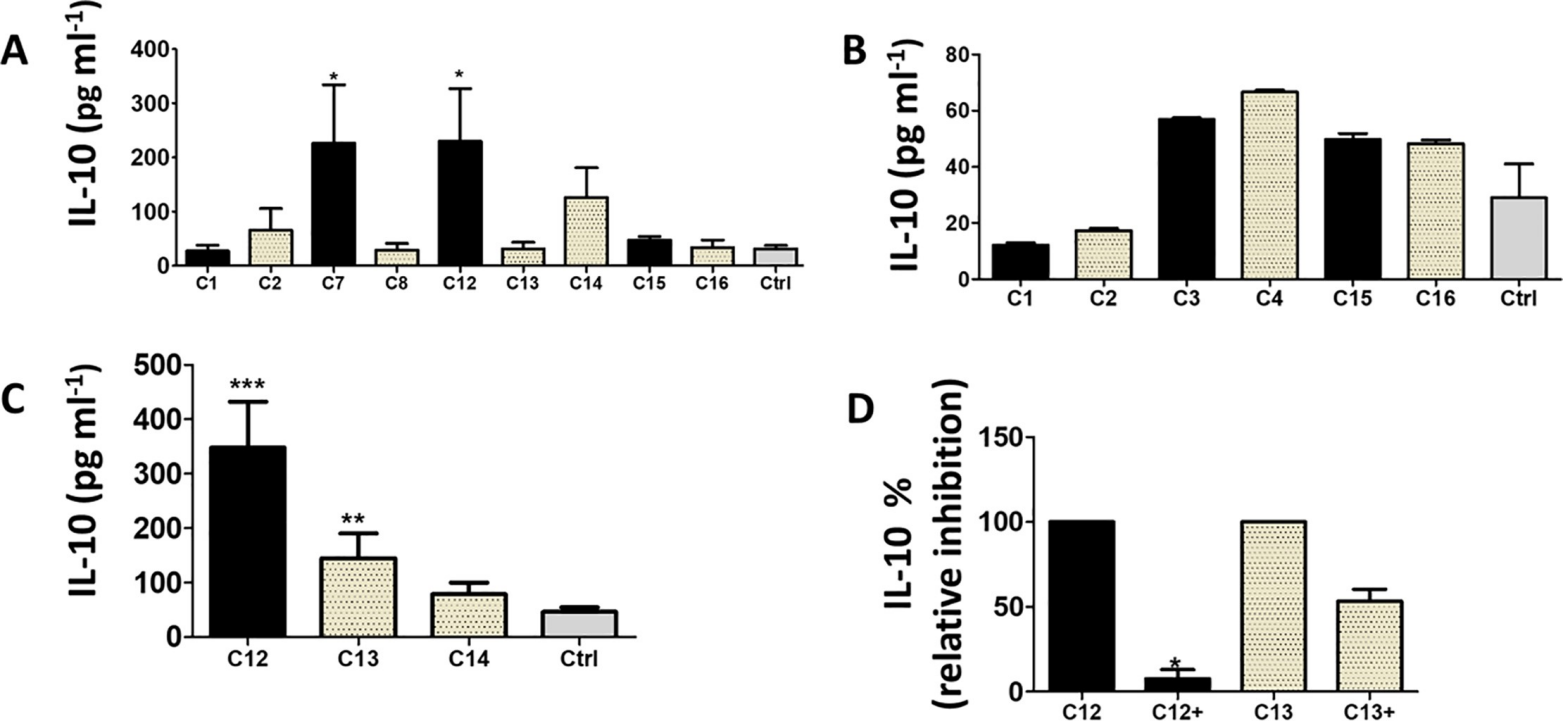
Envelope stimulation of MDDC IL-10 secretion. A) Luminex assay was used to measure the concentration (pg ml^-1^) of IL-10 released by 7 donor MDDCs in response to pseudovirus (PSV) C1, C2, C7, C8, C12-C16 and levels were compared using Mann Whitney two tailed comparison. B) IL-10 ELISA was used to measure the concentration (pg ml^-1^) of IL-10 released by 4 donor MDDCs in response to PSV C1, C2, C3, C4, C15 and C16 and levels were compared using Mann Whitney test. C) Purified recombinant gp140 (C12-C14) were used to stimulate seven donor MDDCs and the secretion of IL-10 was measured by Luminex assay. Concentration of IL-10 was compared between the clones using Wilcoxon signed rank test. D) Three donor MDDCs were stimulated with PSV (C12 and C13) after pre-incubation with recombinant DC-SIGN (+) and IL-10 levels relative to uninhibited MDDC stimulation (%) were compared by paired t test. C1 & C2, C3 & C4, C7 & C8, C12, C13 & C14 and C15 & C16 are Env pairs from the same patient at transmission and chronic infection, respectively.

IL-10 secretion varied 32-fold between clones (range = 21 to 680 pg ml^-1^) and 340-fold between donors (range = 2 pg ml^-1^ to 724 pg ml^-1^). C7, C12 and C14 stimulated MDDC IL-10 release 10 -, 9—and 6—fold, respectively (Fig 2A). The levels of IL-10 induced by C7 and C12 PSV were significantly higher than background (*P* = .04 and *P* = .013), although only C12 remained statistically significant when we adjusted for multiple comparison using the step-down false discovery method. C15 also induced more IL-10 than its CI pair, C16 although this did not reach statistical significance. On the contrary, when CAP177 C3 and C4 IL-10 induction was measured by ELISA (Fig 2B), the CI PSV induced the release of slightly more IL-10 than C3 although this difference was not significant. The relative differences in the ability of C1, C2, C15 and C16 to induce IL-10 secretion measured using IL-10 ELISA was similar to that of the Luminex Assay indicating that the variation in IL-10 release was not an artifact of the method used. Therefore, overall, for the majority of participants infected with a single variant at transmission the TF induced MDDCs to secret higher levels of IL-10 than that of its matched CI variants.

### Role of Env-DC-SIGN interactions in MDDC Interleukin-10 secretion

We next evaluated the capacity of recombinant gp140 to induce IL-10 secretion. As we wanted to confirm the importance of Env in stimulating MDDCs to release IL-10, we selected clones from the same participant that differed significantly in the ability to induce IL-10 secretion. When three purified recombinant gp140 (rgp140) clones, C12, C13 and C14, were tested, we observed a 7 -, 6—and 3—fold increase in IL-10 secretion above that of the background. C13 gp140 was a better inducer of MDDC IL-10 secretion than C13 PSV (Fig 2C), although this variation between assays was not apparent for all clones as TF C12 was the best inducer irrespective of whether PSV or gp140 was used. This could be because PSV and rgp140 were produced in HEK 293T and CHO cells, respectively, which might have influenced their N-glycosylation [36]. Apparently, the potential influence of cell type on Env N-glycosylation was clone-specific as PSV and rgp140 of C12 induced MDDCs to secrete high levels of IL-10 consistent with a role for Env in triggering the release of IL-10.

Although Env binds to DC-SIGN via its HM N-glycans, DCs express a number of C-type lectin receptors (CLRs) and toll-like receptors (TLRs) that have also been found to play a role in HIV infection [37,38]. To better understand the role of DC-SIGN in MDDC IL-10 secretion, PSVs were pre-incubated with recombinant DC-SIGN (rDC-SIGN) to block DC-SIGN binding sites before MDDC stimulation. There was a significant decrease in IL-10 secretion in response to C12 PSV after blocking DC-SIGN binding sites (Fig 2D). This suggests that Env binding to DC-SIGN was involved in inducing MDDCs to release IL-10. On the other hand, blocking DC-SIGN binding sites on C13, the CI clone, only reduced IL-10 secretion by approximately 40%. However, as C13 PSV was a weak inducer compared to C12 (Fig 2A) the assay might not have been sensitive enough to detect IL-10 inhibition. Alternatively, the CI C13 might have evolved to be less dependent on DC-SIGN to induce IL-10 secretion. It is possible that rDC-SIGN, when bound to gp120, blocked the attachment of other MDDC mannose-binding receptors but we observed a similar result when inhibiting with a DC-SIGN specific antibody (Manuscript in preparation), suggesting that DC-SIGN might be required for robust release of IL-10.

### N-glycosylation analysis

When we did N-glycosite (www.lanl.gov) analysis of the 18 Env sequences, we found extensive variation in the number (24–33) and frequency of PNGs (5.6–100%). We also discovered that Envs representing viruses isolated at CI were inclined to carry more PNGs than those isolated at acute infection (AI) (*P* = .009), supporting previous data that AI subtype C Envs tended to carry fewer PNGs [14,15]. There was no apparent association between the presence of PNGs at N241, N262, N276, N295, N356, N386, N392 and N448 with IL-10 secretion because although 6/7 sites were conserved in C12, C15, C16, C17 and C18, the PSV of these clones stimulated MDDCs to release highly varied levels of IL-10 (Table 1). This suggests that PNGs previously shown to bind DC-SIGN and enriched with HM on TF Env, are not associated with changes in MDDC IL-10 secretion.

### Role of Env incorporation in robust stimulation of IL-10 secretion

**Fig 3:**
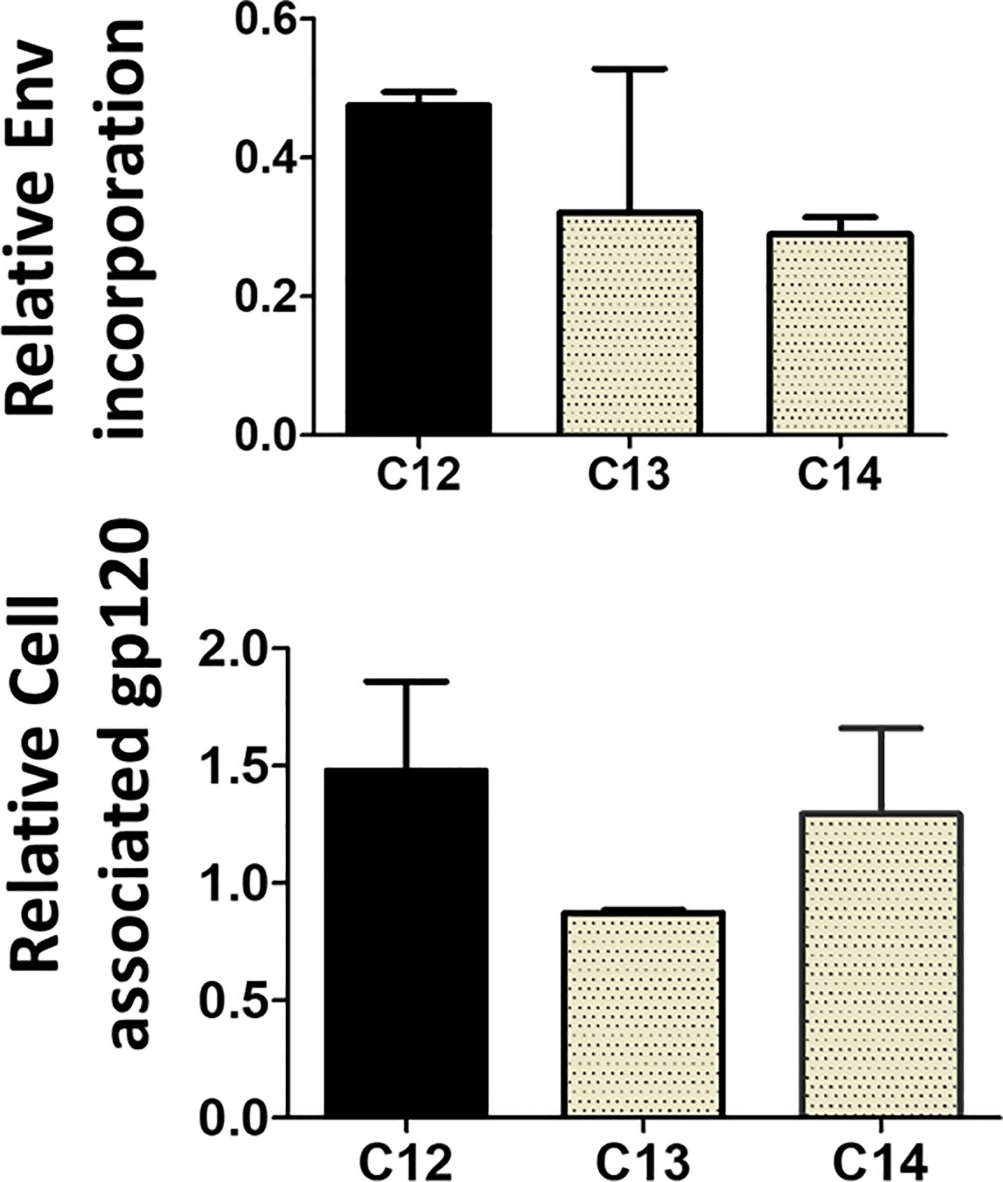
Env expression and incorporation into pseudovirus. HEK 293T cells were co-transfected with pSG3Δenv and C12, C13 or C14 env. The culture medium was harvested and the cells lysed for SDS PAGE and Western blotting. A) Culture medium was ultracentrifuged to concentrate pseudovirus (PSV) and B) 100 μg cell lysate were probed with anti-sheep gp120 antibody. β-actin and p24 were detected as loading controls for Env expression and PSV incorporation, respectively. The average levels of C12, C13 and C14 from two independent experiments were normalised to β-actin and p24 and compared using one-way Anova (Prism 5.0).

The ability of Envs to stimulate MDDCs to release varying levels of IL-10 could be due to how well the glycoproteins are incorporated into PSV. Furthermore, levels of incorporated Env might also be responsible for the difference in PSV entry efficiency, DC-SIGN binding and *trans*-infection between clones (Fig 1). When levels of PSV-incorporated Env were measured by Western blotting and densitometry analysis, the magnitude of the variability in Env incorporation was relatively small in comparison to differences in IL-10 secretion and there was no significant difference between Env incorporation for the matched clones, C12, C13 and C14 (Fig 3A). However, the less efficient incorporation of C14 Env relative to C12 could explain the marginal differences in PSV entry efficiency (Fig 1A) and IL-10 release (Fig 2A) between the two clones. As expression levels might influence the number of Env particles incorporated into virus [39] we also compared levels of cell-associated Env and found that there was also no significant difference in Env expression between C12, C13 and C14 levels (Fig 3B). Furthermore, there were only two mismatches between the sequences of TF (C12) and CI clones (C13 and C14) within the cytoplasmic tail of gp41, the region involved in Env incorporation [39]. It is thus highly unlikely that Env incorporation is driving the ability of Env to induce MDDC’s to secrete different levels of IL-10, suggesting that there might be an alternative mechanism.

### Role of IL-10 in transmission

**Fig 4:**
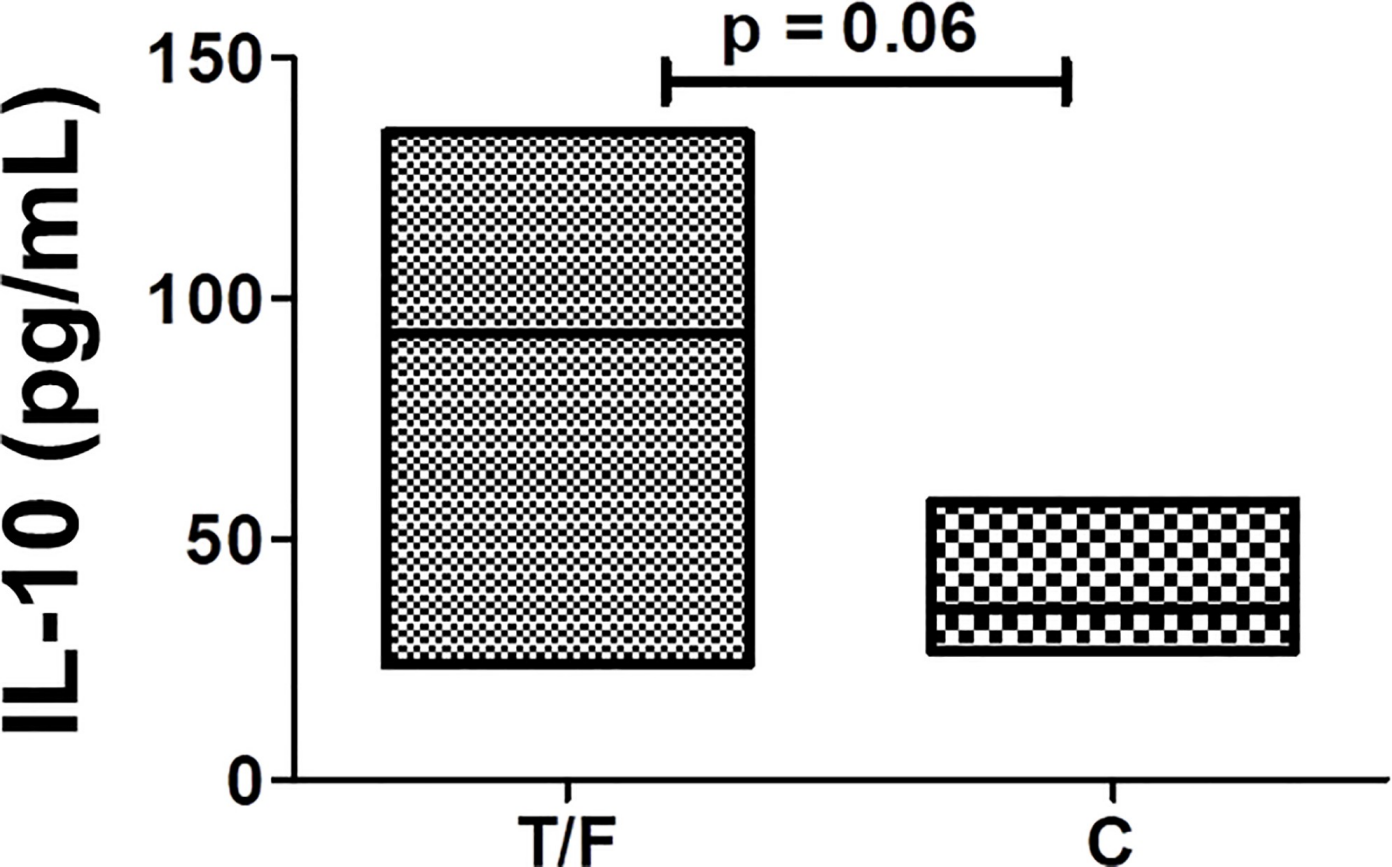
Association between MDDC IL-10 secretion and HIV-1 Transmission. Envelopes (Envs) were grouped on whether they were sampled 2–5 weeks post-infection (wpi) or 2–3 years post-infection (ypi). Pseudovirus was used to stimulate up to seven MDDC donors and median IL-10 levels were compared between matched acute and chronic infection pairs using a Wilcoxon signed rank paired test. Data represents all nine clones used in Fig 2.

We grouped Envs according to time of sampling and found that PSV from 2–5 weeks tended to induce higher levels of IL-10 than those from 2–3 ypi (*P* = .060) (Fig 4). The difference between IL-10 levels released by each MDDC donor in response to matched TF and CI clones were highly variable (Table 2). However, TF PSVs induced most donor MDDCs to release more IL-10 than their matched CI clones. The most robust MDDCs were isolated from donor 5 as the cells released 106.4 –fold more IL-10 in response to C7 than C8. The ability of Env to stimulate MDDCs to release IL-10 is thus highly donor-specific. Overall, these findings suggest that most TF Envs induce DCs to secrete high levels of IL-10, a central regulator of immune responses. The secretion of IL-10 could suppress the ability of DCs to induce an effective adaptive T cell immune response within the FGT and thereby favour HIV survival after transmission.

**Table 2:** Variation in IL-10 secretion by MDDC donors.

	*Change in IL-10 secretion in response to matched TF and CI clones				
Clones	Donor 1	Donor 2	Donor 3	Donor 4	Donor 5
C1/C2	0.7	0.3	0.9	0.7	0.3
C7/C8	23.7	5.5	2.0	7.2	106.4
C12/C13	20.5	5.2	3.2	6.9	42.1
C15/C16	1.2	0.9	1.7	2.7	7.5

*Change in IL-10 secretion is represented as fold decrease calculated as the ratio: IL-10 induced by the transmitted founder clone/IL-10 induced by the matched CI clone

## Discussion

Despite numerous studies, it is still not known what makes one HIV-1 variant more transmissible than another [40,41]. Although sequence analysis indicated that transmission of a single variant was not a random, stochastic event [42], identification of a transmission motif common to TFs remains elusive. However, Env N-glycosylation has been consistently linked to HIV-1 transmission over a number of studies [18,43–47]. More recently, a PNG at position N415 was found to be significantly under-represented in Env sequences from AI viruses compared to those from CI [48]. Other studies went one step further and suggested that TF gp120 PNGs are enriched with HM/H N-glycans [16], the very N-glycans involved in binding to DC-SIGN of DCs localised to the sub-mucosa of the FGT [49]. Virus-bound-DCs then migrate to lymph nodes and infect CD4+ T cells via infectious synapses [8,17,50]. Overall, these studies suggest that Env N-glycosylation might play a very important role in HIV-1 transmission due to its interaction with DC-SIGN. In this study, we sought to determine whether PSV entry efficiency, binding to DC-SIGN and *trans-*infection might provide TFs with an advantage during HIV-1 transmission and/or whether subtype C Env played an alternative role in enhancing HIV-1 transmission through regulation/suppression of DC function.

We found that TF Envs were not significantly better than CI clones at mediating CD4+ dependent TZM-bl entry, DC-SIGN binding and *trans*-infection of CD4+ cells, confirming results of a previous study that also used matched TF and CI PSV albeit from subtype B variants [51]. Overall, our data suggested that Envs’ ability to infect CD4+ cells, either directly or indirectly via *trans*-infection, does not advantage variants during transmission.

DCs are vital players in directing both innate and adaptive IRs and some viruses have been shown to deregulate DCs to promote their own survival [8]. We found that Env induced MDDCs from healthy donors to release IL-10 and levels of cytokine varied from donor to donor and between Envs, supporting previous findings [9]. However, the majority of MDDCs sampled randomly from healthy donors secreted relatively higher levels of IL-10 in response to TF than CI Envs. Nasi *et al*. (2017) reported that HIV-1 was able to modulate DC cytokine responses and this ability varied according to viral strain [6]. Therefore, both virus and host genetics contribute to the extent to which DCs release IL-10. Furthermore, we confirmed that Env binding to DC-SIGN was important for the secretion of IL-10 and we therefore hypothesised that the more DC-SIGN-Env interactions, the more cytokine released. However, incorporation of C12 Env into PSV was not significantly higher than C13 and C14, suggesting that the extent to which Env is incorporated does not drive the stimulation of DC-SIGN-mediated IL-10 secretion.

Instead our data suggested that if DC-SIGN was important then Env N-glycosylation could be a determining factor in the deregulation of DC immune responses. However, when we compared the frequency of PNGs known to bind DC-SIGN and/or carry HM residues to the ability of PSV to stimulate IL-10 release, there was no apparent association. It is possible that other unidentified Env PNGs are also important for interacting with DC-SIGN and triggering the release of IL-10. Although we did not identify why certain Envs have the ability to induce MDDCs to release IL-10, we have shown that TF Envs, cloned from participants infected with a single variant, stimulated the release of higher or equal levels of IL-10 than their matched CI controls irrespective of MDDC donor. Therefore, variants that interact with DCs via DC-SIGN and stimulate the release of IL-10 might be selectively transmitted. In mice, IL-10 was also found to be critical for the establishment of persistent lymphocytic choriomeningitis virus (LCMV) infection [52–54]. This could be because pathogens or pathogenic products that induce IL-10 expression could subvert DC function to favour immune suppression and thus pathogen survival [55–57].

The total number of bound DC-SIGN does not seem to be important for the release of IL-10 seeing as some Envs were very poor inducers of IL-10 secretion despite binding DC-SIGN (example C1) or conversely, very good inducers of IL-10 with very poor binding to DC-SIGN (example C14). Instead, DC-SIGN might be able to bind similar recognition patterns that trigger different signalling pathways leading to other IRs [58]. Alternatively, slight changes in binding affinity between Env and DC-SIGN might initiate cell signalling of different intensity. DC-SIGN binds N-glycans in multiple ways depending on α1-2-linked mannose content and the different modes of interaction significantly alter binding affinity [23]. It is possible that TF Envs are enriched with α1-2-linked mannose that enhance binding affinity to DC-SIGN initiating a more intense signal and increased IL-10 secretion. Envs carry multiple forms of oligomannose structures comprising five to nine mannose residues with the latter structures binding with much higher affinity to DC-SIGN [23].

HIV infection is associated with genital tract inflammation [59–61] likely due to the high number of target cells in the genital tract fuelling HIV replication [62–64]. Enhanced levels of IL-10 in cervico-vaginal lavages were not associated with inflammation and increased risk of HIV-1 infection in a previous study [65]. Our results suggest that TF Env induces DCs in the female genital mucosa to release high levels of IL-10 which seems contrary to findings of Masson *et al*. (2015). We hypothesise that Env-induced release of IL-10 might be highly localised resulting in autocrine deregulation of DC maturation, emergence of DCregs, inhibition of T cell function [7] [12], and tolerogenic DCs [13]. This localised, focused action might subvert the function of DCs and T cells within the FGT, allowing for viral survival [55,57,66,67]. However, the actual mechanism whereby TFs stimulate DCs to secrete high levels of IL-10 and how this may possibly lead to viral persistence in the local environment and establishment of infection requires further investigation. More detailed comparison of the type of HM N-glycans present on Envs from matched AI and CI variants could confirm whether TF Env are enriched with α1-2-linked mannose N-glycans that bind to DC-SIGN with higher affinity.

In conclusion, we have shown that TF Env tend to stimulate MDDCs to release high levels of IL-10 via DC-SIGN. As DC-SIGN-Env binding is important for this process, PNGs might form a motif that drives MDDC IL-10 release. Identification and targeting of this motif by vaccines might reduce localised IL-10 levels in the FGT caused by HIV infection and prevent deregulation of DC immune responses after transmission.

## Supporting information

S1 Fig Correlation analysis of Envelope functions. Spearman correlation was used to determine the association between *trans-*infection using Raji-DC-SIGN cells with A) entry efficiency and C) DC-SIGN binding; between *trans-*infection using MDDCs and B) entry efficiency and D) *trans-*infection using Raji-DC-SIGN cells. Correlation analysis was done using GraphPad Prism 5.0.
(TIF)
