# Supplementary figures and images for: Selective transmission of some HIV-1 subtype C variants might depend on Envelope stimulating dendritic cells to secrete IL-10
Source: PLoS One. 2020 Jan 24;15(1):e0227533. doi: 10.1371/journal.pone.0227533 (PMC6980567; doi:10.1371/journal.pone.0227533)

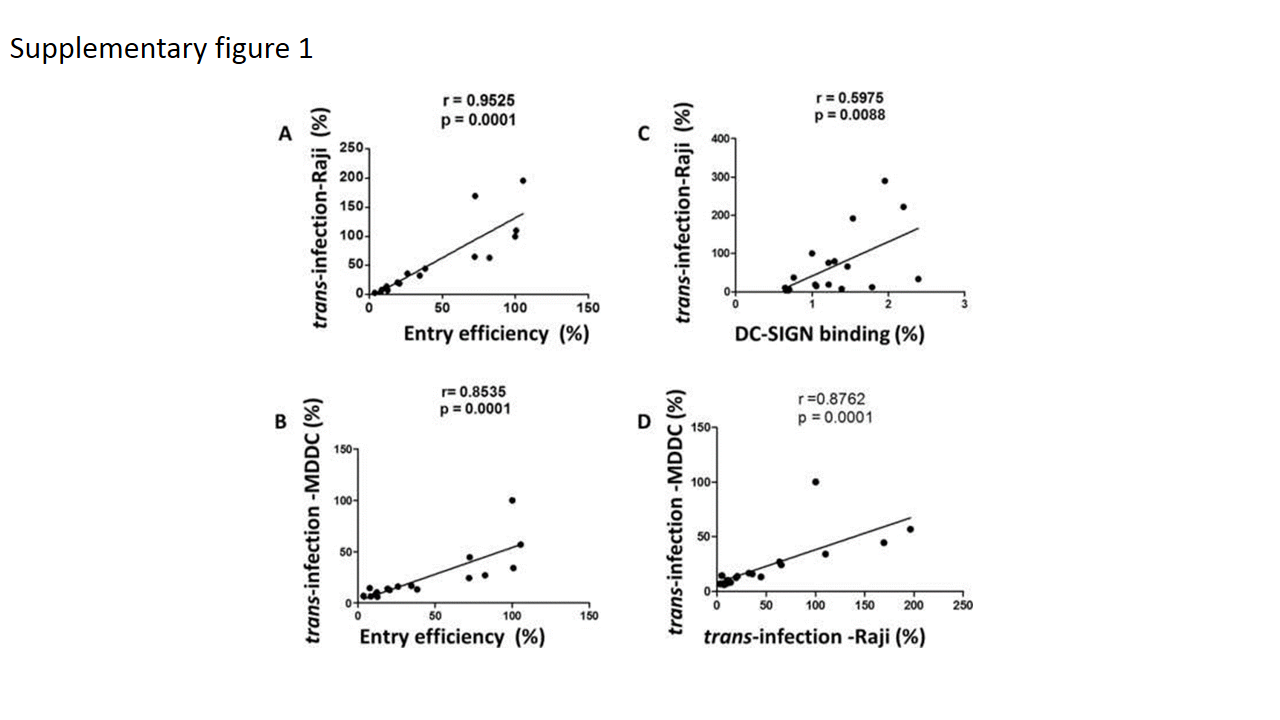

Supplement: S1 Fig — Spearman correlation was used to determine the association between trans-infection using Raji-DC-SIGN cells with A) entry efficiency and C) DC-SIGN binding; between trans-infection using MDDCs and B) entry efficiency and D) trans-infection using Raji-DC-SIGN cells. Correlation analysis was done using GraphPad Prism 5.0. (TIF) [file pone.0227533.s001.tif]
